# Person-Environment Fit and Employee Creativity: The Moderating Role of Multicultural Experience

**DOI:** 10.3389/fpsyg.2018.01980

**Published:** 2018-11-01

**Authors:** Kaiqing Wang, Yijie Wang

**Affiliations:** ^1^Department of Sociology, Jiangsu Normal University, Xuzhou, China; ^2^Department of Sociology, Hohai University, Nanjing, China

**Keywords:** person-environment fit, demands-abilities fit, needs-supplies fit, employee creativity, multicultural experience

## Abstract

Previous research has demonstrated the positive effects of congruent personal and environmental characteristics on creativity. None of them, however, has tested the formal theory of person-environment fit for predicting creativity in the context of multicultural experiences. This study examined the effects of two versions of person-environment fit (Demands-abilities fit and Needs-supplies fit) on employee creativity in China, taking into account the moderating role of multicultural experiences. The results, based on the data of East Asian Social Survey in the Chinese General Social Survey (CGSS) in 2015, showed employees with demands-abilities fit have lower creativity than those with demands-abilities misfit; nevertheless, the demands-abilities fit creates a growing impact on employee creativity with increasing multicultural experience. Additionally, the higher the needs-supplies fit, the stronger the employee creativity; and, the needs-supplies fit creates a growing impact on employee creativity with increasing multicultural experience. It shows that different versions of person–environment fit have different effects on employee creativity and multicultural experience moderated the effects of person-environment fit on employee creativity. Implications for research and practice are discussed.

## Introduction

With the development of economic globalization and modern science and technology, the world economy is marching from the era of industrialization toward that of knowledge economy, and the economic growth is increasingly driven by technological innovation. In this context, creativity has become dramatically pivotal to companies aspiring to gain advantages in an ever-increasingly competitive market environment (e.g., Amabile, 1988; Woodman et al., 1993; Shalley and Gilson, 2004; George and Zhou, 2007; Mumford, 2011). This is not only embodied in the fact that creativity is crucial to an organization’s economic success (e.g., Geroski et al., 1993; Eisenhardt and Tabrizi, 1995), but that creativity conduces to improved organizational performance, such as crisis response capability (Tushman and O’Reilly, 1996), organizational planning capability (Mumford et al., 2008), teamwork spirit, and organizational citizenship (Amabile et al., 2004). Individual creativity has been the cornerstone and origin of creativity at all levels (Amabile, 1988; Woodman et al., 1993). For organizations, employee creativity plays a prominent role in organizational innovation and boom (Amabile, 1996; Shalley et al., 2004). On that account, it is of paramount significance for the survival and boom of companies to probe into driving factors in employee creativity and its mechanism of action so as to motivate employee creativity, which has also become a principal topic for scholars.

Early research on creativity mainly focused on personalities (e.g., Barron and Harrington, 1981), cognitive processes (e.g., Sternberg, 1988; Schooler and Melcher, 1995), lifecycle rules, and individual factors (e.g., Simonton, 1990; Gardner, 1993). Nevertheless, creativity is not completely individualized but rather a function of individual and contextual factors (e.g., Woodman et al., 1993; Amabile, 1996; Amabile and Pillemer, 2012). An increasing amount of recent research has begun to explore creativity from the perspective of the interaction between individual factors and contextual ones (e.g., Oldham and Cummings, 1996; George and Zhou, 2001).

Most researchers usually select certain variables from individual and contextual factors to construct interaction terms, to which the approach can discover the relationship between specific variable combinations and employee creativity, but can hardly explain the relationship between creativity and the interaction between individuals and contexts as a whole (Wang and Sun, 2010). However, the viewpoint of person-environment fit (P-E fit) provides us with a path to explain the interaction between individuals and contexts at large (Livingstone et al., 1997). As the kernel of Organizational Behavioral Science, this viewpoint refers to the consistency, matching, and similarity between the person and the environment (see Edwards, 2008, for a review). Many studies have concentrated on the relationship between P-E fit and its types and employee attitudes and behaviors (e.g., Kristof-Brown et al., 2005b; Oh et al., 2014; Milliman et al., 2017), yet few has paid attention to P-E fit and employee creativity, and even few have focused on the positive impact of objective matching on employee creativity (e.g., Livingstone et al., 1997; Choi, 2004b; Du and Wang, 2009; Wang and Sun, 2010; Zhang and Long, 2013). In such case, what impacts will employees’ perceived fit create on creativity?

People in different sociocultural environments may present diverse creative expression patterns (Kharkhurin, 2010). In other words, a specific sociocultural environment leads to discrepancies in employee creative activities (e.g., Simonton, 1975; Csikszentmihalyi, 1999; Chiu and Kwan, 2010). Many scholars have analyzed the differences in individual creativity under different cultural backgrounds from a cross-cultural perspective (e.g., Lubart, 1999; Paletz and Peng, 2008). This cross-cultural perspective holds that, due to different internal structures of diverse cultures, the unity of culture has been exaggerated, whereas the heterogeneity of culture has been ignored; therefore, focusing merely on the comparison of cultural similarities and differences has overlooked the inter-cultural interaction and dynamic process (Morris, 2014). In that way, nowadays with more and more diversified forms and approaches of cultural interaction, how will people’s multicultural experience affect the relationship between person-environment and employee creativity? This is also one of the questions demanding an answer in this study.

## Literature Review and Hypotheses

### Research on Person-Environment Fit

Contemporary P-E fit research is often traced to Parsons (1909) who developed a matching model to describe the fit between attributes of the person and characteristics of different vocations. Afterward, Murray’s need-press model and Lewin’s field theory lay a theoretical foundation for the research on P-E fit (see Edwards, 2008, for a review). For a long time, P-E fit has been discussed from the two perspectives of supplementary fit and complementary fit (Muchinsky and Monahan, 1987). Supplementary fit usually means that individuals and organizations have similarities in terms of goals, attitudes and values; for example, individuals and organizations deem that autonomy is of greater significance (Kristof, 1996). Complementary fit denotes that the resources owned by the individual or the organization are able to meet each other’s needs; for example, the skills possessed by the individual meet the requirements of the organization, or the resources provided by the organization meet the needs of the individual (Cable and Edwards, 2004). That being the case, complementary fit can be divided into demands-abilities fit and needs-supplies fit (Caplan, 1987; Edwards, 1991; Kristof, 1996). Demands-abilities fit considers fit from the angle of the individual meeting the requirements of the organization, that is, the matching between the person and the organization occurs only when the individual has abilities the organization needs. Organizational requirements generally include job requirements, role expectations, organizational norms, and certain aptitudes, skills, time, and energy owned by the individual. Needs-supplies fit considers fit from the angle of the organization satisfying the requirements of the individual, that is, the matching between the person and the organization emerges only when the organization meets needs and preferences of the individual. Individual needs generally include innate biological and psychological needs, values, and achievement motives. Organizational supply refers to satisfying individual needs through internal and external resources such as food, money, social participation, and self-realization opportunities (e.g., Edwards et al., 1998; Cable and Edwards, 2004).

A large number of P-E fit studies center on changes in employee attitudes, physical and psychological responses, and behavior brought by various types of fit (see Kristof-Brown and Guay, 2011 for a review), such as job satisfaction (e.g., Livingstone et al., 1997), organizational commitment (e.g., Kristof-Brown et al., 2005a), organizational identification (e.g., Cable and DeRue, 2002), and work stress (e.g., Livingstone et al., 1997; Edwards and Shipp, 2007). As a result variable of P-E fit, creativity has also been attached importance by some researchers (Schneider et al., 1995; Tierney et al., 1999). One standpoint holds that person-environment interaction will lead to P-E fit, going with which the homogeneity of the person will enhance as well; yet, the homogeneity may impede the divergent thinking of employees, thus undermining their creativity (Schneider et al., 1995). Another standpoint holds that P-E fit brings good psychological feelings to employees, which in turn leads to improvements in employee creativity (Tierney et al., 1999). It can thus be told that the relationship between P-E fit and creativity is inconclusive. Given that supplementary fit usually involves both the individual and the organization, and that this study places stress on the impact of employees’ perceived fit on their creativity, this article looks back at length on the relationship between creativity and demands-abilities fit as well as needs-supplies fit, based on which research hypotheses have been put forward.

### Demands-Abilities Fit and Employee Creativity

Considering that knowledge and skills are very crucial or fundamental antecedent variables in individual creativity (Amabile, 1988; Woodman et al., 1993; Ford, 1996), the kernel of demands-abilities fit tends to emphasize that the knowledge and skills of employees fulfill the requirements of their jobs (Edwards, 1996). In other words, knowledge and skills provide a set of cognitive pathways that individuals can follow to resolve a given problem or accomplish a given task (Amabile, 1988). The higher the employee’s demands-abilities fit, the more knowledge and skills they acquire to satisfy job requirements; hence, it is easier for them to get rid of their conventional thinking modes. The multidimensional cognitive structure facilitates employees to leverage knowledge and skills more flexibly at work, thus constantly developing new ideas and put them in to practice (Boon et al., 2011).

On top of that, in accordance with the Social Cognitive Theory (Bandura, 1986), demands-abilities fit can motivate individuals to construct positive self-recognition during the work process and promote them to develop inherent driving forces based on implicitness and abilities (Lee and Antonakis, 2014). Integrating self-efficacy with creativity theory, Tierney and Farmer (2002) proposes the concept of creative self-efficacy, which refers to the individual’s belief in his ability to creatively accomplish a specific task, embodying his confidence in demonstrating creative abilities and behavior during the work process. When individuals have the abilities to address specific problems and accomplish specific tasks, or when their capabilities exceed the requirements of the job, they believe that they can successfully adopt innovative thoughts and ideas to solve problems at work. On the contrary, when individuals’ own abilities are unable to meet job requirements, they show little confidence in creativity (Choi and Price, 2005). Good self-efficacy stimulates creativity (Choi, 2004a). It can thus be seen that demands-abilities fit may produce an indirect effect on creativity through positive self-cognition (e.g., Choi and Price, 2005; Du and Wang, 2009; Wang and Sun, 2010; Zhang and Long, 2013). On account of the above analysis, Hypothesis 1 has been proposed.

Hypothesis 1: Employees with demands-abilities fit have greater creativity than those with demands-abilities misfit.

### Needs-Supplies Fit and Employee Creativity

Needs-supplies fit indicates whether the resources provided by the organization can meet the needs of employees. When the resources provided by the organization are the same as the resources employees expect from the organization, needs and supplies match up with each other (Edwards, 1996). In line with the Social Cognitive Theory, when the resources provided by the organization comply with individual needs of employees, they hold the organization is creating a positive working environment, under which they tend to have higher commitment and loyalty to the organization, and give organizations corresponding rewards based on the principle of exchange and reciprocity, thereby stimulating individual creativity (Wang and Sun, 2010). In addition to the principle of reciprocity, the perception of fairness in social exchanges also exerts an influence on employee creativity. When employees think that their efforts and rewards match, they will show more creativity (Janssen, 2000).

From the perspective of Motivational Theory, when employees’ needs are met by the organization to a higher degree, they will have a lasting emotional input in work, show more sense of responsibility in the work process, and are willing to step up efforts, in order to fulfill the psychological needs of autonomy in internal motivations. Proactive work motives make them more willing to think hard in the work process and therefore more likely to pose new problems and come up with new solutions (Farzaneh et al., 2014).

A more painstaking study divides needs-supplies fit into two categories of internality and externality by its nature (Cable and Edwards, 2004). Internality refers to the matching between achievement motivation, self-realization, and values; whereas externality usually refers to material incentives provided by the organization. From this perspective, different aspects of needs-supplies fit produce diverse effects on creativity. When employees can obtain some resources from organizations, such as autonomy and decision-making power, they will express a higher level of creativity. In such research needs-supplies fit is internal (e.g., Shalley et al., 2000). Yet conclusions are inconsistent regarding the relationship between external material incentives and employee creativity (Zhou and Shalley, 2003). The Humanistic Psychology School represented by Amabile believes that external incentives inhibit internal motivation and creativity (Amabile et al., 1986, 1996); on the contrary, the Learning School represented by Eisenberger holds that external incentives promote internal motivation and creativity (Eisenberger and Rhoades, 2001; Eisenberger and Aselage, 2009). Upholding the ideas of freedom and individualism, Amabile et al. (1986, 1996) deems that: mankind’s nature lies in the pursuit of freedom, self-expression, and self-realization; external motivation destroys employees’ interest in creativity and causes employees to lose their sense of self-determination, resulting in employees paying close attention to short-term results and incentives themselves, but no longer trying new solutions to problems. As a consequence, the Humanistic Psychology School regards external incentives as being inherently destructive for employee creativity to explore new discoveries. In contrast, upholding utilitarianism, Eisenberger and Rhoades (2001) and Eisenberger and Aselage (2009) believes that through reasonable material incentives, individuals can effectively enhance their self-determination and performance pressure, thereby improving internal motivation and creativity. Be that as it may, new research revealed that external motivation is also conducive to inspiring creativity, indicating that external motivation and internal motivation can synergistically influence creativity (Shalley and Zhou, 2008). The above analysis leads to Hypothesis 2.

Hypothesis 2: The higher the needs-supplies fit, the higher the employee creativity.

### The Moderating Role of Multicultural Experience

Culture is “any knowledge legacy shared and passed down in a community that can satisfy the psychological needs of individuals or communities” (Chiu et al., 2010). The multicultural perspective breaks through the “trait theory” of cross-cultural perspective (see Peng et al., 2017 for a review). Viewing culture as an implicit sharing of knowledge among individuals, it delves into how a particular situation activates the cultural constructs in individuals’ brain and influences their mentality and behavior. From this perspective, researchers begin to center on the relationship between multicultural experience and creativity (e.g., Chiu and Hong, 2005; Leung et al., 2008; Maddux et al., 2010). As pointed out by existing studies, multicultural experience can raise the level of individual creativity by improving the epiphany learning, long-distance imagination and concept formulation of individuals, increasing the extraction of unconventional knowledge, and generating new ideas through unfamiliar cultures (Leung et al., 2008). Multicultural experience facilitates individuals to encode information in different ways, to draw new concepts and ways of thinking from other cultures, and to establish multiple connections among concepts (Maddux et al., 2010). Such being the case, individuals draw new ideas from different cultures and integrate them in novel ways when in problem-solving scenarios. Integrating seemingly irrelevant concepts in different cultures conduces to the expansion of conceptual category in the brain (Chiu and Hong, 2005). In brief, the individual creativity can be increased by multicultural experience which exposes individuals to novel concepts and ideas, and enhances their abilities to perceive, process, and arrange cultural information.

As to employees with richer multicultural experience, when their perception ability fulfills job requirements, multicultural experience enables them to encode information in various manners, to draw new concepts and ways of thinking from other cultures, to get rid of conventional thinking modes, thus constantly forming and applying new ideas to the practical problem-solving process.

When the resources provided by the organization meet the needs of employees, needs and supplies match up with each other. For employees with richer multicultural experience, their ability to integrate diverse cultural, together with the proactive work motivation brought by needs-supplies fit, can better enhance their problem-solving creativity. The following hypotheses are thus put forward:

Hypothesis 3: The impact of demands-abilities fit on employee creativity increases with employees’ multicultural experience.

Hypothesis 4: The impact of needs-supplies fit on employee creativity increases with employees’ multicultural experience.

## Materials and Methods

### Data Collection Procedure

Chinese General Social Survey (CGSS) is the first nationwide, comprehensive, and continuous academic survey in China. CGSS system collects data from multiple levels of society, community, family, and individual, summarizes the trends of social change, and dives into topics of major scientific and practical significance.

Similar to former surveys, the CGSS sampling method in 2015 adopted a multi-stage, stratified probability sampling design, which, at the level of village, employed the sampling method based on map addresses that has been generally recognized by large-scale domestic social and economic surveys. The 2015 CGSS field survey put to use the Ominisurvey questionnaire survey system for the first time, drastically raising the timeliness and quality of data collection. The survey covered 478 villages in 28 provinces/cities/autonomous regions across China and completed 10,968 valid personal questionnaires.

In addition to the core module, the 2015 CGSS project also included the work module of East Asian Social Survey (EASS), which had an average probability of 1/6. The EASS work module in CGSS 2015 was applied to analyze the relationship between P-E fit and employee creativity and completed a total of 1,743 valid questionnaires. As P-E fit questions in the data were only for non-agricultural workers, this study chose samples of “currently engaged in non-agricultural work” for analysis according to the work experience in the questionnaire. After removing the cases with missing data, a number of 543 valid questionnaires were obtained.

### Measurement

#### Person-Environment Fit

P-E fit measurement contains direct measurement and indirect measurement (Kristof, 1996). Direct measurement is conducted by the fit degree perceived by the individual, such as “My abilities meet the needs of organizational development”; while indirect measurement includes crossover and individual methods. The former involves the measurement of two objects – the individual and the organization, as well as the statistics of fit degree; the latter uses different questioning methods to put questions only to the individual, such as “I often have new ideas” and “My company is filled with dense innovation atmosphere,” and then analyzes the match between individual creativity and organizational creativity atmosphere.

Both direct and indirect measurement has pros and cons (Kristof, 1996). Indirect measurement can produce a separate and meaningful demonstration of the internal psychological process of comparison between the person and the environment (Edwards, 1994). However, it is possible that people who make intuitive judgments about a subject rather than going through a process of actual comparison and the subject in question (Choi and Price, 2005). In comparison, though failing to discriminate between independent effects of the individual and the environment and subject to potential response bias, direct measurement can obtain unique information that indirect measurement cannot (Choi and Price, 2005), and in fact it has been considered more effective than indirect measurement (Cable and Judge, 1997). This study thus employs the direct measurement of P-E fit. This measurement method means that employees directly report the level of matching they perceive; moreover, in whatever circumstances, the matching is achieved as long as the individual perceives it (Kristof-Brown et al., 2005b).

The indicators for the measurement of demands-abilities fit are: “Whether you think the education you received matches the needs of your current job” and “Whether you think the skills you gained match the needs of your current job.” Options for the questions are cross-combined to form “Both fit,” “Either fit,” and “Neither fit.”

For the measurement of needs-supplies fit, it has been divided into two categories of internality and externality by its nature. The conclusion on the relationship between internality fit and creativity is relatively consistent, whereas that on between externality fit and creativity is not (Zhou and Shalley, 2003). To verify the relationship between externality fit and employee creativity, this study attaches more importance to externality fit without regard to internality fit for the time being. The questionnaire developed a five-item index (α = 0.930) to obtain participants’ self-reported assessments of their needs-supplies fit. Sample items were “From the perspective of work skills, do you think your company offers you a reasonable salary” “From the perspective of job performance, do you think your company offers you a reasonable salary.”

#### Employee Creativity

Former studies have defined creativity as outcome-oriented or process-oriented. Researchers like Amabile tend to adopt the “outcome-oriented” definition. They define creativity as the creation of newfangled and useful ideas or opinions related to product, service, and process (e.g., Shalley, 1991; Amabile et al., 1996; Ford, 1996; Oldham and Cummings, 1996). By contrast, researchers like Parnes are prone to adopt the “process-oriented” definition. They define creativity as a process in which employees attempt to create new outcomes from behavior, cognition, and emotion, and concentrate on the individual’s creative behavior in complicated situations (e.g., Parnes, 1967; Drazin et al., 1999). In accordance with the orientation of creative process measurement, this study defines employee creativity as the creative behavior of employees when solving problems in daily life as well as work situations.

From the angle of measurement, the measurement of creativity includes subjective evaluation and objective measurement. Subjective evaluation includes self-evaluation, expert evaluation, and superior evaluation (e.g., Shalley, 1995; Zhou, 1998; George and Zhou, 2001; Tierney and Farmer, 2002); while objective measurement involves patent disclosures, technical reports, and ideas submitted to suggestion programs (Shalley et al., 2004). Although each measurement method has its own pros and cons, this study applies the method of employee self-evaluation to measure employee creativity. The reasons are: (1) the employee’s knowledge of job information and perception of their own behavioral motives are more delicate than their superiors; (2) the creativity assessment is highly susceptible to the preference of evaluators, bringing about various understandings and thus vast differences in evaluation results; (3) the superior assessment is easily induced or blinded by the surface behavior of employees good at performance, however, superiors turn a blind eye to the truly creative behavior of some honest employees (Janssen, 2000). In addition, previous research has focused on the creativity of employees in the face of specific tasks; whereas this study focuses on the creativity of employees in entire workdays (Elsbach and Hargadon, 2006), which can be either the creativity in a particular task at work or the creativity in addressing daily problems.

The questionnaire developed a five-item index (α = 0.720) to obtain participants’ self-reported assessments of employee creativity. Sample items were “I often like to try new unusual things” and “When learning new things, I prefer to try my own unique method.”

#### Multicultural Experience

Multicultural experience implies the opportunity to communicate directly or indirectly with foreign cultural elements or members (Leung et al., 2008). It consists of big multicultural experience and little multicultural experience. The former denotes years of experience of living abroad or emigration and thus in-depth comprehension of and exchanges with foreign cultural elements; however, the latter indicates foreign cultural elements exposed to daily life other than emigration or living abroad, for example, the experience of people who have not gone abroad learning foreign languages and watching American dramas (Rich, 2009). Previous studies have laid emphasis on big multicultural experience rather than little multicultural experience. Yet, in today’s globalization, people have more opportunities and easier access to foreign cultural elements, such as language learning, media reading, cultural activities, and consumer behavior (Stürmer et al., 2013), hence little multicultural experience deserves more concern and thus the focus of this study. Since bilingualism is a pivotal indicator for measuring little multicultural experience, bilingual immersion can develop the individual’s multicultural identity and promote them to better comprehend multiculturalism, which is a tool to measure multicultural experience (Bialystok, 2001). Bilingualism in the article refers to the two languages of Chinese and English. Due to a large number of dialects in China, the dialects will no longer be subdivided here, but Mandarin and dialects are considered as a whole. The English ability hence represents the level of bilingualism. The questionnaire developed a five-item index (α = 0.966) to measure employees’ bilingualism. Sample items were “What do you think of your English listening ability?” and “What do you think of your English speaking ability?”. The higher the total score is, the stronger the bilingual ability, and the richer the multicultural experience. Variables description is shown in Table 1.

**Table 1:** Descriptive variables (*N* = 543).

Variable	Percent	Mean	*SD*	Minimum	Maximum
**Gender**					
Male	59.85%				
Female	40.15%				
**Education level**					
Primary school or lower	9.58%				
Junior high school	32.41%				
High school	24.49%				
College and higher	33.52%				
**Demands-abilities fit**					
Both fit	67.59%				
Either fit	16.94%				
Neither fit	15.47%				
Age		39.57	11.365	18	75
Needs-supplies fit		3.41	0.840	1	5
Multicultural experience		1.87	0.955	1	5
Employee creativity		3.14	0.860	1	5


## Results

### Demands-Abilities Fit and Employee Creativity

In this study, stepwise linear regression analysis has been used to verify relevant hypotheses. Based on the procedure in stepwise linear regression analysis suggested by Baron and Kenny (1986), the first to input into the regression equation is control variable; the second is the variable of demands-abilities fit (this variable is a three-category variable and thus converted into two dummy variables) and multicultural experience; the third, the interaction between demands-abilities fit and multicultural experience.

Model 2 and model 3 in Table 2 reflects the impact of demands-abilities fit on employee creativity. Including merely control variable, Model 1 indicates gender and age are negatively correlated with employee creativity. In other words, women’s creativity is lower than men’s (*B* = -0.168, *P* < 0.05); the creativity becomes lower with age (*B* = -0.018, *P* < 0.001). With demands-abilities fit and multicultural experience added in, Model 2 shows employees whose education and skills both match the requirements of the job have lower creativity than those not (*B* = -0.218, *P* < 0.05), and the same with those either of whose education or skills match the requirements (*B* = -0.288, *P* < 0.05), which is the opposite of Hypothesis 1; Multicultural experience exerts a dramatic impact on employee creativity (*B* = 0.122, *P* < 0.05), that is, the richer the multicultural experience, the higher the employee creativity. Model 2 significantly increases the explanation of employee creativity (ΔR^2^ = 0.021, *P* < 0.05).

**Table 2:** Stepwise linear regression of person-environment fit and employee creativity (*N* = 543).

Dependent variable	Model 1		Model 2		Model 3		Model 4		Model 5	
					
	*B*	*SE*	*B*	*SE*	*B*	*SE*	*B*	*SE*	*B*	*SE*
Female	-0.168^**^	0.072	-0.194^***^	0.072	-0.201^***^	0.072	-0.172^**^	0.072	-0.165^**^	0.072
Age	-0.018^****^	0.003	-0.016^****^	0.003	-0.015^****^	0.003	-0.015^****^	0.003	-0.016^****^	0.003
**Education (primary school or lower = 0)**										
Junior high school	-0.198	0.130	-0.196	0.129	-0.202	0.129	-0.210	-0.207	0.129	-0.207
High school	-0.077	0.136	-0.143	0.137	-0.152	0.136	-0.123	-0.124	0.136	-0.124
College and higher	0.155	0.134	-0.009	0.148	-0.007	0.148	0.000	0.018	0.148	0.018
Multicultural experience			0.122^**^	0.049	-0.091	0.106	0.117^**^	0.049	0.105^**^	0.050
**Demands-abilities fit (Neither fit = 0)**										
Both fit			-0.218^**^	0.095	-0.237^**^	0.095				
Either fit			-0.288^**^	0.123	-0.288^**^	0.124				
Both fit × multicultural experience					0.236^**^	0.109				
Either fit × multicultural experience					0.326^**^	0.149				
Needs-supplies fit							0.105^**^	0.042	0.111^***^	0.042
needs-supplies fit × multicultural experience									0.082^*^	0.044
Constant	4.130^****^	0.226	4.328^****^	0.236	4.327^****^	0.235	3.502^****^	0.290	3.497^****^	0.290
*R*^2^	0.109		0.130		0.139		0.130		0.135	
Adjusted *R*^2^	0.101		0.117		0.123		0.118		0.122	
ΔR^2^	0.109^∗∗∗∗^		0.021^∗∗^		0.009^∗^		0.021^∗∗^		0.006^∗^	
*F*	13.141		9.976		8.622		11.372		10.427	


*^∗^*P* < 0.1, ^∗∗^*P* < 0.05, ^∗∗∗^*P* < 0.01, and ^∗∗∗∗^*P* < 0.001.*

Model 3 takes into account the interaction variable between demands-abilities fit and multicultural experience, which is highly significant, and its coefficient is positive (*B* = 0.236, *P* < 0.05). It is nevertheless implausible to verify the significance of overall interaction simply based on the significance of product term coefficient, because the overall interaction has over one degree of freedom and it depends on whether the R^2^ variation of the main effect model (Model 2) and product term model (Model 3) is remarkable or not. The result exhibits that R^2^ variation changes dramatically (ΔR2 = 0.009, *P* < 0.1). This implies that as long as an additional unit is included in multicultural experience, the average difference in creativity increases by 0.236 between employees whose education and skills match job requirements and whose education or skills match the requirements. In other words, the impact of education and skills matching with job requirements on employee creativity increases with employees’ multicultural experience getting richer. Hypothesis 3 has been therefore validated.

### Needs-Supplies Fit and Employee Creativity

Likewise, the stepwise linear regression analysis has been applied to validate hypotheses related to needs-supplies and employee creativity. The first to input into the regression equation is control variable; the second is the variable of needs-supplies fit and multicultural experience; the third, the interaction between needs-supplies fit and multicultural experience.

Model 4 and model 5 in Table 2 reflects the impact of needs-supplies fit on employee creativity. With needs-supplies fit taken into consideration, Model 4 tells that the higher the needs-supplies fit, the higher the employee creativity (*B* = 0.1050, *P* < 0.05), significantly increasing the explanation of employee creativity (ΔR^2^ = 0.011, *P* < 0.01). Hypothesis 2 hence has been verified.

Model 5 adds in the interaction variable between needs-supplies fit and multicultural experience. The interaction term of between needs-supplies fit and multicultural experience is outstanding and its coefficient is positive, implying that as long as an additional unit is included in multicultural experience, the impact of needs-supplies fit on employee creativity increases by 0.082. In other words, the impact of needs-supplies fit on employee creativity increases with employees’ multicultural experience getting richer.

## Discussion

Differing from former studies, this article has revealed that demands-abilities fit produces a negative impact on employee creativity (Wang and Sun, 2010; Zhang and Long, 2013). Wang and Sun (2010) data analysis of 209 employees and their direct superiors has pointed out that the matching degree between job requirements and employee competence is markedly correlated with creativity. Likewise, Zhang and Long (2013) has also proved that demands-abilities fit exercises an outstanding and positive effect on employee creativity by stimulating their innovation self-efficacy. Their research focuses on creativity in the work process, and the creativity of this article includes both work processes and daily life.

Many past studies implied that employees with a low fit lacked sufficient motivation to improve their professional skills by indirect imitation and learning, thus failing to gain more experience needed for more creativity (Shalley et al., 2004; Zhang and Long, 2013). That being the case, when demands and abilities mismatch, does the employee necessarily go through frustration, low self-esteem, indifference, cognitive disorder, or even disharmony (Chatman, 1991) which in turn inhibits creativity? Livingstone et al. (1997) has discovered a U-shaped relationship between demands-abilities fit and pressure. When the individual’s perceived ability falls short of or exceeds the job requirements, the pressure increases; conversely, when the individual’s ability matches the job requirements, the pressure is the minimum. That is to say, demands-abilities misfit puts employees under considerable pressure. Despite that, pressure is not always a conundrum (LePine et al., 2005; Podsakoff et al., 2007), because pressure exposes competitive disadvantages to the individual and forces them to employ new ideas and procedures to solve problems (e.g., Hon et al., 2013). Being indicators of abilities, education and skills have always been considered as critical factors influencing creativity (Amabile, 1983, 1988; Cohen and Levinthal, 1989), on which the research targets at specific domains or specific tasks, such as art, literature, and music. The measurement of creativity in this study does not involve specific domains or specific tasks, but centering on the employee creativity in entire workdays. Demands-abilities fit is a favorable condition for the individual, but it is in this favorable condition that employees may be more inclined to habitual actions and give up on creative actions (Ford, 1996). On the contrary, when the employee is in a misfit condition, pressure is more likely to inspire employee creativity.

In terms of needs-supplies fit, it turns out that the higher the needs-supplies fit, the greater the employee creativity. When exploring the work context of creativity, Amabile et al. (1996) suggest that the degree to which the individual’s work resources are assigned (Amabile et al., 1996) is positively correlated with creativity, but this hypothesis has not been proven yet. They believe this may be related to the nature of individual needs and work supplies as well as individual motivation (Amabile, 1983). This study delves into the reasonable level of material treatment provided by the organization perceived by employees, which not only includes the degree of fit, but also implies the sense of fairness of employees. The more reasonable the material treatment provided by the organization perceived by employees, the greater the needs-supplies fit on the one hand, and the stronger the sense of fairness of employees on the other. In spite of being external fit from its nature, the material treatment can effectively raise the individual’s self-determination and performance pressure to improve intrinsic motivation and creativity (Eisenberger and Aselage, 2009). Moreover, employees will compare their efforts and rewards. If employees perceive that the treatment provided by the organization is fair, the organization and the individual will form a driving force for social exchange (Masterson et al., 2000), and the employee will work hard to pay back for the organization’s care and trust. In such case, employees may proactively seek creative ideas to improve organizational processes or to develop new products and services (Amabile et al., 1996).

According to some studies, multicultural experience is conducive to promoting creativity (Lee and Kim, 2011; Leikin, 2013). Yet, few have explored the moderating role of multicultural experience in P-E fit and employee creativity. This study has proved that multicultural experience plays a moderating role in the impact of demands-abilities fit and needs-supplies fit on employee creativity. Although the combination of education and skills with organizational requirements is not conducive to stimulating employee creativity, this negative effect is weaker for employees with high multicultural experience and stronger for employees with low multicultural experience. Because employees with high multicultural experience learn new concepts and ways of thinking in other cultures, it is easier to get rid of the inherent thinking patterns, constantly form new ideas and apply them to the practical process of problem solving. When needs-supplies fit, compared with employees with low multicultural experience, employees with high multicultural experience have the ability to integrate diverse cultural. Together with the proactive work motivation brought by needs-supplies fit, They can better enhance their problem-solving creativity. The inspiration drawn from this study to organizational management is: the organization should attach great importance to the creativity of employees with demands-abilities misfit, as well as to the role of little multicultural experience while in a context of deepening globalization, so as to expand employees’ multicultural experience through a variety of approaches.

### Limitations and Future Directions

In spite of some valuable research results obtained, there are some limitations that should not be overlooked in this study. The method of self-evaluation has been employed to measure independent variables (demands-abilities fit and needs-supplies fit), dependent variable (creativity), and moderator variable (multicultural experience), of which the analysis results may be influenced by common method variation. In order to avoid the influence of common method variation, process control and statistical test methods were adopted. In the process control, the unnamed questionnaire method is adopted, and the items measuring different variables are randomly arranged and mixed, and the questionnaire is designed by using the reverse problem. In the statistical test, we used Harman’s one-factor test, all the items in the questionnaire scale were used for factor analysis, and 5 factors were extracted from the principal component analysis results when the rotation was not rotated, Total Variance Explained is 69.635%. The first factor explained the variation was 25.415%, and the common method variation was not serious. More methods will be used to avoid the influence of common method variation in the future. Due to data limitations, this study has probed into the relationship between personal-environment fit, multicultural experience, and employee creativity from a multicultural perspective. However, multicultural experience is measured only from a bilingual perspective, and it will be measured in multiple dimensions in future. In addition, as pointed out by Zhao Zhiyu et al., “The research on cultural and social psychology has undergone the paradigms of cross-cultural psychology, cultural psychology, multicultural psychology, as well as polycultural psychology emphasizing the influence of intercultural relationships on psychological processes” (Chiu et al., 2013). Under the polycultural psychology paradigm, culture mixing has taken culture and psychology research a step forward. Scholars have carried forward the research tradition of multicultural psychology and have a profound understanding of the forms and categories of intercultural interactions and their social, cultural and psychological consequences thus caused (Morris et al., 2015). On this account, the next-step research should be carried out on the impact of cultural mixing on employee creativity and its influencing mechanism.

## Conclusion

In compliance with the complementary perspective of person-environment fit, this study has dived into the relationship between demands-abilities fit, needs-supplies fit and employee creativity, as well as the impact of multicultural experience on the relationship. It has been revealed that, unlike previous research findings, employees with demands-abilities fit have lower creativity than those with demands-abilities misfit; nevertheless, the demands-abilities fit creates a growing impact on employee creativity with increasing multicultural experience. Additionally, the higher the needs-supplies fit, the stronger the employee creativity; and, the needs-supplies fit creates a growing impact on employee creativity with increasing multicultural experience.

## Data Availability Statement

The datasets (GENERATED) for this study can be found in the (Chinese National Survey Data Archive) (http://cnsda.ruc.edu.cn/).

## Ethics Statement

An ethics approval was not required as per institutional guidelines and national laws and regulations because no unethical behaviors existed in this study. The data of this study were collected by questionnaire survey through a professional service institute. The content of the questionnaire was not involved with any ethical problem. All respondents were informed of the aims of this research and it was indicated that they approved this study when they filled out the questionnaire. For these reasons, the authors considered that we did not need to provide ethics approval and written informed consent.

## Author Contributions

YW organized the database. KW performed the statistical analysis and wrote the first draft of the manuscript. All authors contributed to conception and design of the study, manuscript revision, and read and approved the submitted version.

## Conflict of Interest Statement

The authors declare that the research was conducted in the absence of any commercial or financial relationships that could be construed as a potential conflict of interest.The handling Editor declared a shared affiliation, though no other collaboration, with one of the authors YW.
